# Impact of a nutrition education intervention on nutrition-related self-efficacy and locus of control among women in Lesotho

**DOI:** 10.3389/fpubh.2023.1060119

**Published:** 2023-03-23

**Authors:** Mamotsamai Ranneileng, Mariette Nel, Corinna May Walsh

**Affiliations:** ^1^Department of Nutrition and Dietetics, University of the Free State, Bloemfontein, South Africa; ^2^Department of Biostatistics, University of the Free State, Bloemfontein, South Africa

**Keywords:** Lesotho, nutrition education, self-efficacy, locus of control, women

## Abstract

**Introduction:**

Lesotho is one of the poorest countries in the world with high levels of food insecurity and malnutrition. The aim was to evaluate the impact of a nutrition education intervention informed by self-efficacy and locus of control theories among women in Lesotho.

**Methods:**

A randomized pre-test-post-test design was adopted to implement a systematically designed nutrition education intervention in women from Maseru and Berea districts in Lesotho. Women from selected villages were randomly assigned to comparison and intervention groups. Baseline and post assessments were conducted before, and 6 months after the intervention. Nutrition-related self-efficacy and locus of control were assessed using a semi-structured knowledge, attitudes, beliefs, and practices (KABP) questionnaire.

**Results:**

At baseline, 444 women aged 19–60 years were included. After the intervention, 259 women in the comparison (*n* = 105) and intervention groups (*n* = 154) were interviewed. Self-efficacy beliefs that improved significantly in the intervention group but not in the comparison group included increased confidence that they could eat a healthy diet every day [95% CI for the percentage difference (61.5; 76.7)]; an improved ability to secure several healthy foods in the home; increased confidence in engaging in physical activity [95% CI (29.5; 46.6)]; reducing the amount of salt they used in food [95% CI (2.1; 14.0)]; and compiling a budget for food purchases [95% CI (56.1; 72.1)]. Regarding locus of control, the belief in a personal capacity to take charge of one's health through the production and consumption of healthy food improved in the intervention group [95% CI (12.4; 25.0)] but not in the comparison group [95% CI (15.9; 0.4)]. At follow-up, a significantly larger percentage of participants in the intervention group also believed that they could take control of their health and that they could prevent some illnesses by the food they eat.

**Conclusion:**

A nutrition education intervention that is systematically planned and framed on selected theories of health behavior improved nutrition-related beliefs in self-efficacy and locus of control.

## 1. Introduction and background

Supporting food and nutrition security is undoubtedly the first and foremost strategy to ensure access to safe, acceptable and adequate sources of food (1). However, a complex set of issues influence people's eating patterns and behavior. These factors include, among others; inadequate knowledge of what to eat; social, socio-economic and cultural factors; as well as psychological factors that determine food choices (2).

Lesotho is a small mountainous country with a population of 2.2 million people. It is considered to be one of the world's least developed and poorest countries, with half of the population living in poverty and many exposed to high levels of food insecurity (3–7). The urban transition is characterized by migration to urban areas from remote rural areas that has contributed to rapid urban population growth. Food production has declined due to several reasons, including climate change, lack of arable land, soil erosion, Basotho farmers' inability to compete with cheaper imports of food and the high prevalence of HIV (4). All of these factors have a direct impact on food security in Lesotho and emphasize the importance of implementing relevant, sustainable and culturally acceptable interventions.

.evious studies in Lesotho have shown that Basotho women lack nutrition-related knowledge and that their dietary intake is largely inadequate. As a result, their nutritional status is challenged and they present with high levels of malnutrition, including both undernutrition, overnutrition, and micro-nutrient deficiencies (8–10). Although undocumented nutrition-related initiatives have been undertaken in Lesotho (including radio talks and presentations at women's groups), most of these are fragmented and focused on single topics (8).

Theories of health behavior have popularly been used in health education and promotion interventions (11–14). These theories are designed to address many things, including understanding why individuals behave the way they do; correcting misinformation (11, 12); identifying barriers to change; and promoting culturally relevant messages and interventions (13, 14).

Self-efficacy, derived from Bandura's Social Cognitive Theory (13, 15) has been used as both a theory and a construct in other theories. Self-efficacy theory proposes that for individuals to change a specific behavior that might be difficult to do or need a lot of effort to accomplish, they must believe in their capacity to initiate and perform the change (16) and must have confidence that they can overcome the barriers to performing the behavior (17). Self-efficacy may be affected by prior failed attempts at performing a particular behavior and this might affect an individual's self-esteem, which in turn may affect self-efficacy (18). It may also be affected by the effort required to perform the behavior (17). Self-efficacy is recognized as an important determinant in the transition from one stage of readiness to another in the stages of change model (17, 18) and in the perception of barriers as described in the health belief model (18).

Locus of control is the personal control that people feel toward health. It is either internal or external. An internal locus of control means that a person believes that they can influence their health, while external locus of control means that an individual believes that their health is in the hands of others (16). Individuals with a strong internal locus of control are more likely to practice behavior that prevents illness and promotes health, such as eating a prudent diet and seeking more information about health, than those with an external locus of control (16).

The bases for using self-efficacy and locus of control as framework theories for the current nutrition education intervention was largely influenced by the assertion that for an individual to change their dietary and other health behaviors, they need the motivation to want to change. The most effective method of raising motivation to change is restoring self-esteem and self-efficacy and developing an internal locus of control (19).

Given the above, the present study aimed to determine the impact of a nutrition education intervention, framed on improving self-efficacy and internal locus of control, on the nutrition-related behavior of Basotho women. To our knowledge, no systematically designed nutrition education intervention programmes that are relevant to the unique situation of women in Lesotho have previously been implemented.

## 2. Methods

### 2.1. Development of the intervention

The research team designed a nutrition education intervention based on identified needs of women in Lesotho. An in-depth literature review was conducted to identify knowledge, beliefs, attitudes, and practices that promote or hinder desirable nutrition actions in resource-limited settings. The South African Food-Based Dietary Guidelines (SAFBDG) and selected theories of health behavior—in this case, self-efficacy and locus of control—were used as a framework for the intervention. Although it is acknowledged that environmental mediators and barriers play an important role in health behaviors (social determinants of health), the current intervention was intended to address person-related barriers and mediators that could be addressed within the resource-limited environment that the women live. Based on this, culturally appropriate intervention goals and content were developed to respond to the identified needs by creating awareness, cultivating self-efficacy and internal locus of control, and providing information to increase knowledge.

The intervention was compiled in accordance with health psychology and health promotion guidelines of theories of health behavior and had the following characteristics: It was systematically designed as it followed steps and sequence and used strategies that included questioning and brainstorming (11). It built social capital as it promoted cohesion and built capacity among the women to support each other in healthy eating (12). The intervention was presented during workshops in a group work setting to ensure that all participants could participate actively in discussions and develop personal skills for effective decision-making related to specific nutrition needs (11, 13). This interaction that encouraged the gaining of knowledge and skills in an independent and empowering environment (20). Pictures and flip charts were used to build, analyze and discuss the content and printed take-home messages were provided to participants.

### 2.2. Design, population, and sample

The current study adopted a randomized pre-test post-test design. Two out of 10 districts, Maseru and Berea, were included in the study because both their urban and rural areas are fairly accessible. Urban and rural areas were defined according to the Lands Surveys and Physical Planning (LSPP) boundaries in Lesotho (6). Randomization was done using a sampling frame and every second village was assigned to the intervention group. Separate villages were selected for intervention and comparison groups to prevent spill over or contamination within villages.

All women in the age group 19–60 years who signed informed consent were eligible to participate. The choice of women in this age group was made with the knowledge that in Lesotho, women are homemakers and decide what is to be eaten in the family. The following categories of women were excluded from the study: domestic workers that lived in the homes of women participating in the study, pupils/students, acutely ill and disabled women, and pregnant and lactating women. These groups have unique characteristics that need special considerations that were beyond the scope of this study.

### 2.3. Techniques and procedures

Ethics approval was obtained from the Health Sciences Research Ethics Committee of the University of the Free State and the Ministry of Health in Lesotho. Baseline and follow-up assessments were carried out during 2013 at baseline and 6 months after the intervention.

After a random selection of villages to be included in the study, the researcher arranged information sessions with the community leaders/counselors to sensitize them to the intended project and obtain approval. The community leaders were asked to call *pitsos* (community gatherings; *pitsos* are the most effective method of disseminating information to the communities in both the urban and rural areas in Lesotho), where information related to the project was disseminated. Women were invited to participate in the study, after which arrangements for data collection and implementing the nutrition education intervention were made.

Five research assistants (qualified nutritionists from the National University of Lesotho) were recruited and trained to conduct the interviews. The pilot study was completed among 26 volunteers in an area other than Maseru and Berea, and thus these women were not included in the main study. The pilot study served to determine if questions were understood clearly and the time that it would take to complete an interview.

Volunteers assembled at the agreed venues on the set dates and times arranged for baseline measurements and interviews. After the participants had signed consent, the researcher and field workers completed the questionnaires in a structured interview with each participant. Nutrition-related self-efficacy and locus of control were assessed using a semi-structured knowledge, attitudes, beliefs, and practices (KABP) questionnaire designed by the research team. To assess self-efficacy and locus of control, participants' responses to specific statements were categorized in terms of whether they believed in their own capability to produce and eat a healthy diet; and whether they had an internal or external locus of control regarding food.

To assure validity, the questionnaire was based on an in-depth literature review and compiled in accordance with the standard methods suggested by health psychology and health promotion experts and followed the suggested guidelines of theories of health behavior (11, 12, 16). Reliability of the questionnaires was confirmed on a randomly selected 10% of the participants 1 month after the baseline interviews. The answers obtained on the two occasions were compared, and if an answer to a question differed by more than 20%, the question was deemed unreliable and was excluded from the analysis. Only the question related to income was found to be unreliable and excluded.

The nutrition education intervention was presented after completion of the baseline survey. Ten 2-day participatory workshops were presented in intervention groups (Box 1). One workshop was presented per week, and each lasted about 5 h. Six months later follow-up interviews were conducted in the same groups. The length of time between measurements was kept relatively short to avoid the effect of seasonal differences in availability and accessibility of food, and therefore the impact on eating practices (21), but long enough to adopt sustainable changes (16, 18).

Box 1 **Examples of participatory intervention activities.**

Self-awareness/self-assessment of vulnerability/threat:

- Describe own nutrition and related lifestyle behaviors;
- Describe perceptions of personal vulnerability;
- Describe perceptions of personal threat.

Barriers:

- Identify barriers to achieving a balanced diet [e.g., unavailability, lack of money, lack of land (gardens), time, cost, distance, lack of knowledge, and lack of motivation];
- Identify barriers to physical activity (e.g., time, lack of motivation, lack of knowledge, and lack of facilities);
- Devise ways of dealing with barriers.

Personal control/social norms and cultures:

- Express feelings of personal power/control over barriers;
- Express whether they believed that their health was in the hands of chance/fate/luck/witchcraft (external locus of control) or whether they believed that their health was in their own hands (internal locus of control);
- Identify social norms and cultures that promote or inhibit the performance of recommended action (e.g., the role played by peers, parents, husband, and relatives).

Thoughts and feelings:

- Express thoughts and feelings regarding intended nutritional changes by reporting how the changes are going to affect them personally;
- Predict how their families are going to react to the new changes and how this is going to affect them personally;
- Propose ways to deal with the reactions, both personally and otherwise.

Self-efficacy/goal setting/intention to act:

- Describe confidence in achieving a balanced diet every day/week;
- Demonstrate the ability to compile a budget for food;
- Describe intended changes to food production, purchases, and preparation.

Knowledge:

- Use food pictures to demonstrate which foods are included under the different FBDGs;
- Identify foods that are considered to be healthy and less healthy.

After completion of the interviews, women were served a light meal of sandwiches with tea and fruit.

### 2.4. Statistical analysis

The initial and follow-up assessments were described by frequencies and percentages for categorical data and medians and ranges for numerical data, calculated per group. The change from initial to post assessment was calculated per group and compared by means of 95% confidence intervals.

## 3. Results

At baseline a total of 444 participants (204 in the comparison (C) group and 240 in the intervention (I) group) were included in the study. At follow-up, 105 comparison and 154 intervention participants were interviewed.

The median age of participants was 43 years (range 19–60 years), with 65% of participants in the control group and 52% of those in the intervention group living in a rural area. Only about half of participants had completed primary school education (C =54%; I = 48%) and were in a traditional marriage (C = 47%; I = 48%). Fifty-five percent of participants in the control group and 46% in the intervention group were unemployed. Few participants were employed (C = 18.8%; I = 24.4%), self-employed (C = 11.6%; I = 12.6%), engaged in subsistence farming (C = 1.93%; I = 1.63%), or had a part-time/piece job (C = 6.8%; I = 3.7%). Although the majority of participants owned a house (C = 78.3%; I = 87.0), very few had a bathroom in the house (C =16%; I = 27%), and fewer still had access to their own water tap (C = 8%; I = 14%), with the predominant access to water being a communal tap (C = 62%; I = 57%). Few participants had access to electricity (C = 33%; I = 4I%) and many used a pit latrine system (C = 62%; I =56%). Most had a kitchen/place for cooking (C = 84%; I = 81%) where some used open fire (cow dung, maize sticks, wood) as fuel for cooking (C = 35%; I = 23%). In most households, only one member of the family was employed (C = 71%; I = 63%).

### 3.1. Nutrition-related beliefs informed by self-efficacy theory

At baseline, 30% of participants in both the comparison and intervention groups indicated that they were confident that they could eat a healthy diet every day (C = 32.3%; I = 32.0%). After the intervention, a statistically significant improvement was seen in the intervention group, but not in the comparison group [95% CI for the percentage difference (61.5; 76.7)].

To determine whether or not participants had access to the food to enable them to eat a healthy diet every day, they were asked if they had the following foods in their house: breakfast cereal, rice, maize-meal (ground maize), samp, vegetables, fruits, meat, legumes, milk, eggs, and bread flour (Table 1).

**Table 1:** Changes in nutrition-related self-efficacy.

	**Comparison group**					**Intervention group**				
	**Baseline**		**Follow-up**			**Baseline**		**Follow-up**		
	***n***	**%**	***n***	**%**	**95% CI for % diff**	***n***	**%**	***n***	**%**	**95% CI for % diff**
**I believe that I can eat a healthy diet every day**	64/198	32.3	28/104	26.9	−5.7; 15.6	78/244	32.0	148/155	95.5	61.5; 76.7^*^
**I have the following foods in my house:**										
Breakfast cereal	33/199	16.6	26/105	24.8	0.3; 13.3^*^	53/245	21.6	100/155	64.5	36.3; 52.1^*^
Rice	72/199	36.2	41/105	39.1	−3.6; 1.7	94/245	38.4	94/155	60.7	17.9; 32.2^*^
Maize-meal	198/199	99.5	104/105	99.1	−2.7; 5.2	241/245	98.4	154/155	99.4	−2.1; 3.8
Vegetables	174/199	87.4	99/105	94.3	−3.6; 7.8	225/245	91.8	149/155	96.1	−1.0; 8.0
Fruits	77/199	38.7	41/105	39.1	−5.6; 5.6	106/245	43.3	124/155	80.0	34.2; 50.4^*^
Beans	137/199	68.8	77/105	73.3	−1.2; 8.9	158/245	64.5	145/155	93.6	25.5; 40.7^*^
Milk	70/199	35.1	43/105	41.0	−6.0; 2.2	110/244	45.1	116/155	74.8	26.2; 42.2^*^
Eggs	107/199	53.8	66/105	62.7	−4.2; 4.2	148/244	60.7	131/155	84.5	19.9; 34.7^*^
Bread flour	121/199	60.8	70/105	66.7	−0.9; 6.7	136/235	57.9	139/154	90.3	28.7; 45.5^*^

^*^Indicates a statistically significant improvement.

At baseline the majority of participants indicated that they had maize-meal (C = 99.5%; I = 98.4%); vegetables (C = 87.4%; I = 91.8%); and dried beans (C = 68.8%; I = 64.5%) in their houses. After the intervention, availability of bread flour improved significantly from 57.9 to 90.3% in the intervention group [95% CI (28.7; 45.5)] but not in the comparison group.

At baseline, about 40% of participants in both the comparison and intervention groups reported having milk in the house. After the intervention, availability of milk remained more or less the same in the comparison group [95% CI (−6.0; 2.2)] but improved significantly to 75% in the intervention group [95% CI (26.2; 42.2)].

Other significant improvements in the availability of foods that were seen in the intervention group included fruits [95% CI (34.2; 50.4)]; breakfast cereal [95% CI (36.3; 52.1)]; and rice [95% CI (17.9; 32.2)].

To further establish self-efficacy, participants were asked to indicate their confidence in performing certain actions related to eating and other lifestyle factors (Table 2).

**Table 2:** Changes in health-related actions.

	**Comparison group**					**Intervention group**				
	**Baseline**		**Follow-up**			**Baseline**		**Follow-up**		
	***n***	**%**	***n***	**%**	**95% CI for % diff**	***n***	**%**	***n***	**%**	**95% CI for % diff**
Remove fat from meat	58/199	29.2	28/105	26.7	−2.5; 2.5	66/245	26.9	85/152	55.9	21.0; 3.9^*^
Engage in physical activity for 30 min or longer at least 3 times a week	50/199	25.1	19/105	18.1	−10.4; 0.6	52/245	21.2	95/154	61.7	29.5; 46.6^*^
Try to reduce the amount of salt in food	154/199	77.4	77/105	73.3	−7.7; 3.8	213/244	87.3	146/155	94.2	2.1; 14.0^*^
Compile a budget for food purchases every month	76/198	38.4	41/104	39.4	−3.3; 3.3	82/245	33.5	150/155	96.8	56.1; 72.1^*^
Compile a list of foods to buy every week	21/198	10.6	13/104	12.5	−3.3; 3.3	26/245	10.6	110/155	71.0	46.9; 65.4^*^
Compile a list of foods to buy every month	75/198	37.9	44/104	42.3	−5.1; 1.3	95/245	38.8	151/155	97.4	48.9; 65.4^*^

^*^Indicates a statistically significant improvement.

At baseline, almost all items except growing vegetables (C = 88.9%; I = 80.4%) were reported by < 40% of participants. After the intervention, improvements were observed in all items in the intervention group. These included removing fat from meat before cooking [C = 95% CI (21.0; 39.0)]; engaging in physical activity for 30 min or longer at least three times a week [95% CI (29.5; 46.6)]; reducing the amount of salt in food [95% CI (2.1; 14.0)]; and compiling a list of foods to purchase every month [95% CI (48.9; 65.4)] and every week (46.9%; 65.4%).

### 3.2. Nutrition-related beliefs in locus of control

To assess whether participants had an internal or external locus of control regarding food, they were asked to indicate whether or not they agreed with several statements informed by the theory (Table 3).

**Table 3:** Changes in health-related locus of control.

	**Comparison group**					**Intervention group**				
	**Baseline**		**Follow-up**			**Baseline**		**Follow-up**		
	***n***	**%**	***n***	**%**	**95% CI for % diff**	***n***	**%**	***n***	**%**	**95% CI for % diff**
I believe that illness and health are caused by luck, fate or chance	135/198	68.2	69/104	66.4	−5.2; 2.9	134/245	54.7	2/155	1.3	−59.0; −42.9^*^
I believe that illness and health are caused by witchcraft	23/198	11.6	6/104	5.8	−5.2; 2.9	16/245	6.5	1/155	0.7	−10.1; −1.1^*^
I believe that I can take control of my health	115/198	58.1	72/104	69.2	−3.8; 7.6	163/245	66.5	150/100	96.8	16.0; 28.1^*^
I believe that I can prevent some illnesses by the food I eat	140/198	70.7	75/104	72.1	−15.9; 0.4	183/245	74.7	155/155	100.0	12.4; 25.0^*^

^*^Indicates a statistically significant improvement.

At baseline, the majority of participants indicated that they believed that illness and health were caused by luck, fate or chance (C = 68.2%; I = 54.7%), while most indicated that they did not believe that illness and health were caused by witchcraft (C = 64.1%; I = 73.1%). More than half believed they could take control of their health (C = 58.1%; I = 66.5%), while more than 70% reported that they could prevent some illnesses by the food they ate (C = 70.7%; I = 74.7%).

After the intervention, the belief that illness and health were not caused by luck, fate or chance improved significantly in the intervention group [95% CI (−59.0; −42.9)]. In the intervention groups, significant improvements were also observed in the belief that illness and health were not caused by witchcraft [95% CI (−10.1; −1.1)]; that they could prevent some illnesses by the food they ate [95% CI (12.4; 25.0)].

## 4. Discussion

The Basotho women included in the current study were characterized by low levels of education and unemployment. The lack of amenities such as own sanitation, running water and electricity are evidence of the poverty that is endemic to Lesotho. The widespread limited food availability and access in Lesotho (4) have negatively affected food utilization in terms of food choices, preparation and storage. These factors highlight the importance of interventions that empower women to make the best use of available resources.

In terms of self-efficacy, the intervention was successful in improving the confidence of women to eat a healthy diet every day. Before the intervention, hardly 40% indicated that they had confidence in their ability to do so. The nutrition education delivered to the intervention group thus seemed to reaffirm the women's confidence in the foods that they should eat. In framing this intervention on a social cognition model, the study was consistent with other studies that determined the effectiveness of dietary interventions to promote healthy eating using psychological based interventions (22, 23).

Before the intervention, almost all women reported that they had maize-meal in their homes, while about 90% had vegetables (more than 80% grew vegetables in their gardens), leaving little room for improvement due to the nutrition education intervention. On the other hand, improvements were seen in the availability of dried beans, milk, fruits, breakfast cereal, and rice. These improvements are evidence that the nutrition education intervention improved self-efficacy in making healthy food choices. For any health behavior to be performed effectively, individuals must believe in their competency to successfully perform the behaviors in question (16) and must have confidence that they can overcome the barriers to performing the behavior (17).

Although most participants in the current study grew and ate vegetables, most studies report a low intake of vegetables. Nutrition education interventions to improve fruit and vegetable consumption report varying levels of effectiveness, despite most people knowing that fruit and vegetable consumption is positively associated with health benefits (22). In the US, high self-efficacy was associated with consuming fruit and vegetables to prevent chronic disease (24). Self-efficacy has also been used in fruit consumption studies in the Netherlands (25, 26) and in the Middle East (27) and was found to be highly predictive of fruit-eating behavior. Self-efficacy is considered an important factor in the perception of barriers to performing health protective behavior (18). In the current study, growing food reduced perceptions of cost and lack of food as a barrier to eating a healthy diet.

At baseline, < 40% of all women had self-efficacy in removing fat from meat before cooking; in engaging in physical activity for 30 min or longer at least three times a week; and in reducing the amount of salt they used in food. After the intervention, women in the intervention group's self-efficacy to perform health-promoting behaviors relating to food in all these items improved significantly. Similarly, high self-efficacy was found to predict an intention to choose and consume low fat and low sodium foods among North American students (28).

Before the intervention, very few women in the current study compiled a budget or planned food purchases in advance. After the intervention, women in the intervention group were no longer purchasing food every day, indicating that they had learned to plan food purchases. In contrast, the comparison group was not enabled to change this behavior. Improvements in the ability to shop using a list indicate growth in self-efficacy about the importance of budgeting, planning and making healthy food choices.

Generally having high self-efficacy reduces stress levels (28) and motivates compliance with dietary recommendations (29). Availability of food in the home assures readiness to move from contemplation to action in terms of eating a healthy diet. Self-efficacy is recognized as an important determinant in the transition from one stage to another in the stages of change model (19), and has been found to act as an important component in positively influencing healthy eating (30). When used in combination with other theories, self-efficacy proved predictive of good nutrition behavior (31).

An improved perception of personal control as defined by an internal locus of control is predictive of health protective behavior (20). Eating a healthy diet in order to prevent illness is associated with an internal locus of control (16). In other words, individuals respond to health challenges in the way that applies to their context. In terms of locus of control, a large percentage of participants in the current study indicated that they believed that illness and health are determined by luck, fate or chance, while few believed that illness and health were caused by witchcraft. Most participants believed that they could take control of their health and that they could prevent some illnesses by the food they ate. After the nutrition education intervention, a higher percentage of women in the intervention group gave answers that point to an improved internal locus of control.

Shehu and Mokgwathi found that internal locus of control was predictive of internal resilience and its relationship with physical education among adolescents in Botswana (32). Internal locus of control was also reported to predict positive perceptions about the link between good nutrition and longevity in a study amongst elderly participants in the Western Cape province of South Africa (33).

We acknowledge that the inclusion of two areas that were relatively accessible to the research team may introduce a bias in terms of the representation of women in Lesotho. Although there is a general view that poverty is worse in rural areas, the African Food Security Urban Network (AFSUN) reports that the urban poor in Maseru may be the most food insecure in the country (4). The women that agreed to participate in the study may also represent a specific group within the population, with those that are the most impoverished being less likely to participate. Furthermore, the results are based on self-reported responses and opinions of participants. A concerted effort was made to encourage women to answer truthfully during the informed consent process by explaining that the purpose of the study was not to identify participants who did not do things correctly but to empower them to make sustainable changes within their resource-limited setting. Finally, although the research team took considerable care to develop a valid tool, there is no guarantee that this was achieved, and thus the validity of the tool cannot be assured.

## 5. Conclusion

The findings of our study confirm that a nutrition education intervention that is systematically planned and framed on selected theories of health behavior improved aspects of nutrition-related beliefs in self-efficacy and locus of control. Improved self-efficacy was evidenced by increased confidence in the foods that they should eat and an improved ability to secure healthy foods in the home. The intervention was also able to increase the women's beliefs in their own ability to make decisions about food (internal locus of control), empowering them to eat a healthy diet despite their limited resources.

## Data availability statement

The original contributions presented in the study are included in the article/supplementary material, further inquiries can be directed to the corresponding author.

## Ethics statement

The studies involving human participants were reviewed and approved by Health Sciences Research Ethics Committee of the University of the Free State. The patients/participants provided their written informed consent to participate in this study.

## Author contributions

CW and MR: study conception, design, and draft manuscript preparation. MR: data collection and implementation of intervention. MN: statistical analysis. All authors approved the final version of the manuscript.
